# Enhancement of menaquinone- 7 production through immobilization with hydrogel-based porous membranes

**DOI:** 10.1007/s00253-025-13493-3

**Published:** 2025-05-13

**Authors:** Qiu-Hua Zhang, Zheng Wang, Yao-Qiang Wang, Man-Lu Liu, Hai-Jia Su

**Affiliations:** 1 Beijing Key Laboratory of Bioprocess, and Beijing Advanced Innovation Center for Soft Matter Science and Engineering, Beijing University of Chemical Technology, Beijing, 100029 People’s Republic of China; 2 Brother Research Center, Jiangxi Brother Pharmaceutical Co.,LTD, Jiujiang, 332700 People’s Republic of China

**Keywords:** Immobilization, Polyvinyl alcohol, Menaquinone- 7, *Bacillus*, Fermentation

## Abstract

**Abstract:**

The industrial production of menaquinone-7 (MK-7) by *Bacillus subtilis* has been historically constrained by significant challenges in bioprocess efficiency. To address these limitations, we explored an innovative immobilization strategy utilizing a porous thin-film hydrogel system. Specifically, we developed a novel porous thin-film PVA + B@Ca hydrogel immobilization method that fundamentally transforms cell encapsulation and fermentation dynamics. The comparison between PVA + B@Ca hydrogel immobilized cells and free cells in fermentation demonstrated a significant increase in MK-7 yield from 32.76 ± 1.92 to 48.33 ± 2.92 mg/L, as well as a reduction of the fermentation duration from 48 to 24 h. Additionally, the immobilized cells demonstrated good stability during continuous fermentation, resulting in a space–time yield of MK-7 that increased to 2.0 mg/L·h, which was five times higher than that achieved with free-cell fermentation. Mechanistic insights revealed through microscopic analysis highlight the transformative nature of the hydrogel immobilization: The PVA + B@Ca hydrogel’s porous structure creates a protective microenvironment that mitigates cellular stress and maintains optimal metabolic conditions. These findings represent a paradigm shift in understanding cellular immobilization, demonstrating how strategic encapsulation can fundamentally enhance MK-7 fermentation biotechnology.

**Key points:**

*• A novel hydrogel immobilization method was developed for MK- 7 production*.

*• The use of immobilized cells gave a fivefold improvement in the space–time yield*.

**Supplementary Information:**

The online version contains supplementary material available at 10.1007/s00253-025-13493-3.

## Introduction

Vitamin K2 (VK2) is a crucial fat-soluble nutrient, essential for human health, with menatetrenone- 7 (MK- 7), a notable member of the VK2 family, distinguished by its prolonged half-life (Gröber et al. 2014; Morishita et al. 1999). Recent research reveals VK2 as a key player in sustaining overall health (Vermeer and Braam 2001), impacting nearly every major body system by being centrally or peripherally involved in regulating numerous physiological and pathological processes (Ferland 2012; Harshman and Shea 2016; Weber 2001). These regulatory functions underscore the role of VK2 in preventing and treating a broad spectrum of illnesses (Li et al. 2018; Vos et al. 2012). Despite its benefits, synthesizing biologically active all-trans MK- 7 poses significant challenges (Baj et al. 2016). Conventionally, natural fermentation by microorganisms yields only small quantities of MK- 7 (Ren et al. 2020). To overcome these limitations, significant strides have been made in developing high-yield MK- 7 strains through advanced techniques such as metabolic engineering and modular approaches. However, concerns about the safety of genetically engineered bacteria in food products have prompted regulatory restrictions, thereby complicating the widespread adoption of these innovative production methods. This regulatory environment underscores the need for continued research and development to balance high-yield production with safety and regulatory compliance in the synthesis of this vital nutrient (Gao et al. 2021b; Liao et al. 2021; Xu et al. 2017).

Immobilized cell technology can enhance the stability, reusability, and overall efficiency of the fermentation process by entrapping or attaching cells to a solid support (Gao et al. 2021a; Nedović et al. 2011; Zhu 2007). It offers benefits such as easier separation of cells from the product, potential for continuous processing, and improved control over reaction conditions. These advantages make immobilized cell systems an attractive option for large-scale industrial fermentation and bioprocessing applications (Mahanama et al. 2011a; Vaghari et al. 2015). Most studies on immobilized cell fermentation use porous adsorbent materials or spherical/block-shaped hydrogels for adsorption or encapsulation. For instance, Ebrahiminezhad et al. demonstrated that iron oxide nanoparticles were used to immobilize *Bacillus subtilis* var. natto, and specifically improved MK- 7 fermentation productivity by 15% compared to free cells (Ebrahiminezhad et al. 2016). Moreover, microbial cells recovered from broth using magnetic fields in a bioreactor achieved over 95% recovery.

Integrating in situ cell immobilization and product removal has been shown to minimize process costs for MK- 7 production. Durieux et al. studied malic acid-lactic acid bioconversion by using *Lactobacillus buchneri* as the starting strain (Durieux et al. 2000). In a spherical immobilized cell preparation using alginate beads, the strain exhibited greater acid resistance. Alginate particles consumed 4.6 times more malic acid compared to the control. These in situ adsorption immobilization methods enhance cell load and catalytic efficiency, thereby improving both reaction efficiency and stability. However, the industrial application of these materials is limited by challenges, such as complex preparation processes and low resistance to mechanical agitation. As a result, screening materials that possess high mechanical and chemical stability for MK- 7 fermentation offers an alternative solution to the current challenges faced in MK- 7 production.

In our previous study, we identified a novel MK- 7-producing strain, *Bacillus subtilis* BUCT- 184, from soil samples collected on the Tibetan Plateau (Zhang et al. 2024). However, the MK- 7 yield from using free cells of *B. subtilis* BUCT- 184 was relatively low, which limited its potential for industrial application. Herein, we developed a cell immobilization method utilizing PVA + B@Ca hydrogel in the form of a porous thin film, achieved through the screening and optimization of the hydrogel immobilization process. Fermentation production of MK- 7 using the immobilized *B. subtilis* BUCT- 184 cells led to a yield of 48.33 ± 2.92 mg/L with a halved fermentation duration in a single-batch fermentation process. In addition, continuous fermentation of immobilized cells over eight batches resulted in a stable MK- 7 yield, achieving a space–time yield of to 2.0 mg/L·h that was five times higher than that obtained free-cell fermentation. Finally, we provided possible insights into the intrinsic mechanisms by which immobilized cells enhance MK- 7 production through microscopic and scanning electron microscopy (SEM) analyses.

## Materials and methods

### Strain and culture medium

The MK- 7 producing strain *B. subtilis* BUCT- 184 was isolated and deposited in China Center of Industrial Culture Collection (CICC: 25137). The *B. subtilis* BUCT- 184 strain was cultured in 500-mL baffled flasks containing 100 mL of culture media containing 10 g/L soybean peptone (Aoboxing, China), 5 g/L yeast extract (Aoboxing, China), and 10 g/L NaCl (Sinopharm, China). The flasks were incubated at 37 °C with shaking at 150 rpm for 24 h to facilitate growth in the logarithmic phase. Subsequently, precultures were then inoculated into 100 mL of fresh fermentation media containing 100 g/L soybean peptone, 50 g/L glycerol (Sinopharm, China), 5 g/L yeast extract, and 0.5 g/L K_2_HPO_4_ (Sinopharm, China). The inoculated cultures were then incubated at 37 °C with shaking at 200 rpm for 24 h.

### Immobilization method

Chitosan (Adamas, RG), alginate (Adamas, RG, viscosity 200–500 mPa·s), gelatin (Sinopharm, China), and polyvinyl alcohol (General-Reagent, AR, type 1799) were obtained from suppliers. Chitosan, alginate, and gelatin hydrogels were prepared according to previously reported methods (Gómez-Mascaraque et al. 2014; Kawase et al. 1997; Taha et al. 2008). Preparation of PVA: Under sterile conditions in a clean bench, weigh 8 g of polyvinyl alcohol and add it to 82 g of sterile water. Dissolve and sterilize at 100 °C for 30 min. Allow the temperature to drop to room temperature. Under sterile conditions, add 16 g of wet sludge with a moisture content of 50% and stir rapidly at 600 rpm for 1 h. Seal and place in a − 20 °C freezer overnight. Then, cut into 5 mm × 5 mm squares under sterile conditions. After that, remove it and rinse three times with sterile water for further use. Preparation of PVA + B: Under sterile conditions in a clean bench, weigh 8 g of polyvinyl alcohol and add it to 82 g of distilled water. Dissolve and sterilize at 100 °C for 30 min. Allow the temperature to drop to room temperature. Under sterile conditions, add 16 g of wet sludge with a moisture content of 50% and stir rapidly at 600 rpm for 1 h, then reduce stirring speed to 100 rpm. Apply vacuum at − 0.09 MPa for 20 min to obtain a viscous slurry containing the biomass. Using a syringe, drop the mixture into a 4.5% boric acid sterile solution by mass concentration. Allow it to crosslink and cure overnight. After that, remove it and rinse three times with sterile water for further use. Preparation of PVA + B@Ca (see Figure S1 for details): Under sterile conditions in a clean bench, dissolve 66 g of 12% polyvinyl alcohol aqueous solution at 100 °C, and allow it to cool to room temperature. Add 15.8 g of a 25% calcium carbonate sterile aqueous solution and mix thoroughly. Then add 16 g of wet sludge with a moisture content of 50% and stir well for 30 min. Slowly add 2.2 g of a 4.5% boric acid sterile solution by mass concentration while stirring rapidly at 600 rpm for 1 h. Then reduce the stirring speed to 100 rpm. Apply vacuum at − 0.09 MPa for 20 min to obtain a viscous slurry containing the biomass. Evenly spread the slurry on a smooth metal plate measuring 10 cm × 18 cm, with a coating thickness of approximately 0.5 mm. Place it in a vacuum drying oven at 40 °C for 6 h to dry. Peel off the film, cut it into 5 mm × 5 mm squares, and soak in a 1% sodium chloride solution overnight. Rinse three times with sterile water for further use.

### Fermentation procedures

Continuous batch fermentation of MK- 7 was carried out in 5 L glass fermentation tank (Baoxing, China) using 3 L of culture medium composed of 25 g/L glycerol, 50 g/L soybean peptone, 30 g/L yeast extract, 0.5 g/L K_2_HPO_4_, and 1 g/L MgSO_4_. The fermentation tank was maintained at 37 °C for 24 h, with an agitation speed of 600 rpm and an aeration rate of 2.0 VVM (volume of air per volume of liquid per minute). For each batch, 10% of the fermentation broth containing free and immobilized cell beads was used as the inoculum for the subsequent batch. Fresh sterile medium was then added in one go for fermentation. In this continuous batch fermentation, immobilized beads with a mass ratio of 2% were employed.

### Analytic methods

To 5 mL of the whole culture of *B. subtilis*, 1 mL of hydrochloric acid was added. The mixture was subjected to a boiling water bath for 5 min and then cooled to room temperature. Next, 12 mL of an extraction solution consisting of isopropyl alcohol and *n*-hexane in a 1: 2 (v/v) was added. The mixture was vigorously shaken and allowed to settle for 10 min. Following the settling period, the organic layer was separated, and subjected to nitrogen purge for 30 min to remove any remaining solvents, resulting in a yellow oily product. Prior to analyzing the obtained product by HPLC, it was further extracted using 5 mL of isopropanol. Insoluble materials were then removed by passing the solution through a 0.45-μm polytetrafluoroethylene membrane.

The concentration of MK- 7 was measured using a high-performance liquid chromatography (HPLC) system (UltiMate 3000 model from ThermoFisher scientific, USA) equipped with a photodiode array UV detector. The HPLC column used was the Zorbax SB-Phenyl C18, with a particle size of 5 μm and dimensions of 250 × 4.6 mm (Agilent Technologies Inc., USA). The column temperature was maintained at 35 °C, and the excitation wavelength was set at 254 nm. The mobile phase consisted of 950 mL of methanol and 50 mL of an acetic acid solution (50 mM), and run at a flowrate of 0.8 mL/min. The MK- 7 calibration curve was linear within the concentration range of 0.04 to 8 mg/L, with a correlation coefficient (*R*^2^) of 0.9994.

Cell density was determined by measuring the optical density at 600 nm (OD_600_) using a spectrophotometer (model 7200; Unico Instrument Co., Ltd., Shanghai, China). A known amount (c. 300 mg) of dry membranes was incubated in an excess of distilled water for 24 h. Subsequently, a sponge was used to absorb the surface moisture of the wet membrane. The weight swelling ration of the membranes was determined according to Eq. (1) (Kok et al. 2001).

1 $$water\;content\;\left(\%,\frac ww\right)=\frac{\left[\frac{\mathrm{Ws}-\mathrm{Wd}}{\mathrm{Ws}}\right]}{100}$$

where *W*_s_ is the weight of wet membrane and *W*_d_ the weight of dry membrane. Porositywas measured using a density tester (Quarrz AU- 200 VP, China) following the manufacturer’s manual (Żywicka et al. 2019). Specific surface area was measured using porosity analyzer (OGMASTER 7842, China) according to the corresponding manufacturer’s operating manual (Suryanti et al. 2017). Determination of the specific activity of enzyme *Men*A in the *B. subtilis.* BUCT- 184 cells was carried out according to the previous studies (Suvarna et al. 1998; Xu et al. 2017). The morphology of bacteria in the cell culture medium, both immobilized and resting cells, was observed under a 1000 × microscope after 24 h of cultivation based on Gram staining (Moyes et al. 2009). The SEM analysis of PVA + B@Ca hydrogel immobilized cells was performed on Regulus 8100 (Hitachi, Japan).

## Results

### Screening of MK- 7 immobilization materials

To screen an appropriate hydrogel material, we initially investigated the immobilization of *B. subtilis* BUCT- 184 cells using chitosan, gelatin, alginate, and PVA (Figure S2). The corresponding immobilized cells were used for MK- 7 fermentation, and the results are displayed in Table 1. The chitosan-immobilized cells achieved a maximum MK- 7 yield of 28.56 mg/L after 96 h. It is noteworthy that the chitosan immobilization material dissolved due to its pH-sensitive nature, containing acidic or alkaline groups. Changes in pH within the fermentation environment would lead to ionization of chitosan, weakening hydrogen bonds in the hydrogel network and causing swelling. The use of gelatin-immobilized cells yielded a maximum MK- 7 production of 30.21 mg/L after 72 h. However, while dissolved gelatin provides a carbon source for the bacteria, it also increases the viscosity of the fermentation broth, which can negatively impact MK- 7 production. Additionally, gelatin’s temperature sensitivity causes abrupt changes in its swelling properties at certain temperatures, resulting in volume expansion or contraction. This characteristic, coupled with its influence on the viscosity, leads to immobilized cells with reduced mechanical strength, making them unsuitable for prolonged stirring or agitation. Alginate-immobilized cells achieved a maximum MK- 7 yield of 33.62 mg/L after 72 h, which is an improvement over gelatin-immobilized cells. However, these alginate-immobilized cells experienced partial rupture over time. This stability issue arises because alginate hydrogels are primarily formed through the coordination of sodium alginate with calcium ions creating reversible physical cross-linking points. These cross-links can become unstable when stored in phosphate systems for extended periods, leading to the partial disintegration of the gel matrix. In comparison to chitosan, alginate, and gelatin immobilization, PVA-immobilized cells showed superior performance by maintaining their structural integrity and achieving the highest MK- 7 yield of 48.33 mg/L during fermentation, with a shorter fermentation time (24 h).

**Table 1:** Screening of hydrogel materials for immobilization

Hydrogel material	Max OD_600_/T (h)	Max yield (mg/L)/T (h)	Morphology
Chitosan	11.21/88	28.56/96	Dissolution
Gelatin	18.44/68	30.21/72	Dissolution
Alginate	13.67/72	33.62/72	Fracture
PVA	16.67/24	48.33/24	Intact

### Optimization of PVA-based immobilized method

Subsequently, we investigated the impact of different PVA-based immobilization methods (PVA, PVA + B, and PVA + B@Ca) on the fermentation production of MK- 7 (Table 2). For cells immobilized with PVA, the MK- 7 production reached the maximum yield of 28.66 mg/L at the OD_600_ of 12.85. However, for cells immobilized with PVA and then cured with boric acid (PVA + B), dramatical decreases in the cell density (OD_600_ = 7.4) and MK- 7 yield (14.5 mg/L) were observed. Compared to PVA immobilized cells, the porosity, specific surface area (SSA), and weight swelling ratio (WSR) of PVA + B immobilized cells all decreased. These results indicated the immobilized material formed a denser network structure after cross-linking with boric acid, which might hinder the exchange of substances inside and outside the membrane. Therefore, we sought to increase the porosity of the membrane material by adding calcium carbonate. As shown in Table 2, the introduction of a porous structure formed by calcium carbonate in PVA + B@Ca results in a porosity of 98.7%, enhancing the exchange of nutrients, improving cellular respiration space, and reducing cellular stress responses. Additionally, the specific surface area reaches 19.8 ± 1.37 m^2^/g, facilitating better cell adhesion and more uniform cell distribution, which in turn enhances metabolite interactions. This mechanism creates an optimal microenvironment that supports cell growth and metabolic efficiency, directly translating into increased MK- 7 production efficiency. As expected, fermentation using PVA + B@Ca immobilized cells significantly increased cell density and MK- 7 yield, achieving the maximum yield of 48.33 mg/L at an OD_600_ of 16.67.

**Table 2:** Comparison of different PVA-based immobilized methods

Method	Porosity (%)	SSA (m^2^/g)	WSR (%)	OD_600_	MK- 7 (mg/L)
PVA	71.3	8.5 ± 0.35	467	12.85	28.66
PVA + B	52.4	4.4 ± 0.25	307	7.4	14.50
PVA + B@Ca	98.7	19.8 ± 1.37	258	16.67	48.33

**Fig. 1:**
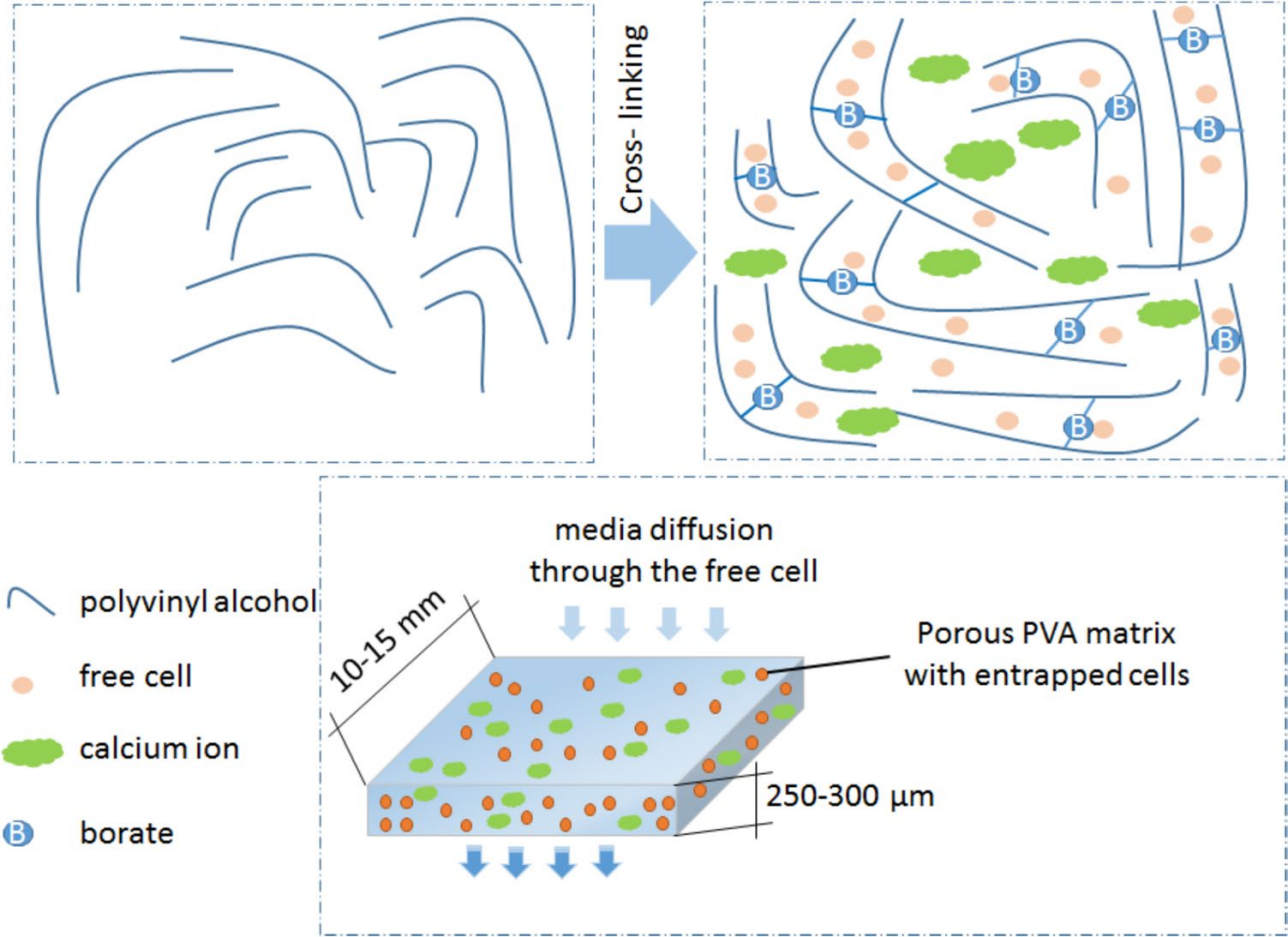
Schematic view of the possible structure of the PVA + B@Ca carrier

Subsequently, we analyzed the possible structure of the PVA + B@Ca carrier and its beneficial effects on enhancing MK- 7 fermentation yield. As shown in Fig. 1, thermal melted PVA forms a soft and elastic polymer with high water content, offering properties similar to natural soft tissue. This characteristic imparts excellent biocompatibility, making PVA an ideal medium for microbial film growth while maintaining high levels of cell activity. When PVA is crosslinked with boric acid, its local molecular arrangement is altered, reducing free volume and increasing the glass transition temperature (Krasňan et al. 2016; Rebroš et al. 2005). This creates an insoluble crosslinked structure that is highly effective for immobilizing cells and controlling the release of active agents and biomolecules. The introduction of calcium carbonate further enhances this system. The introduction of calcium carbonate particles led to the release of CO_2_ and the subsequent formation of numerous pores within the membrane. These pores provide ample space for cell growth and proliferation, enhancing cell production potential. Coating PVA into films with a thickness of 250–300 µm and cutting the hydrogel into evenly sized small particles significantly increases the specific surface area of the hydrogel and minimizes damage from mechanical agitation. This preparation method ensures a robust and stable environment for immobilized cells, suitable for high-efficiency fermentation processes. In addition, the mild operating conditions preserve the original activity of the cells, making this process a valuable immobilization strategy.

### Comparison of single-batch fermentation processes

**Fig. 2:**
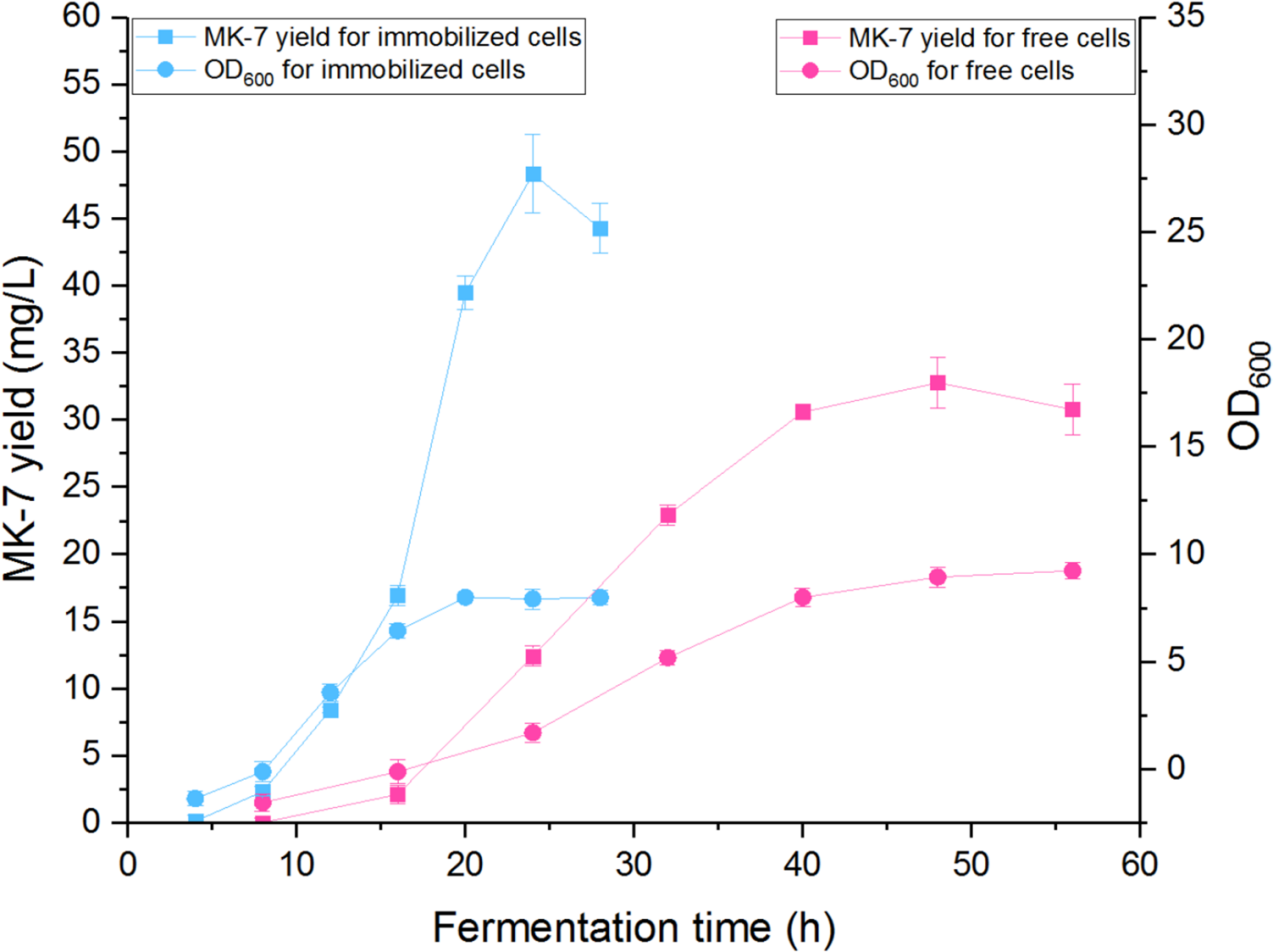
Comparison of yield of MK- 7 and cell density using free cells and immobilized cells in single-batch fermentation processes

The fermentation processes of immobilized cells and free cells were compared and are displayed in Fig. 2. It was observed that the PVA + B@Ca-immobilized cells had a shorter fermentation cycle compared to the free cells. Additionally, the cell density, as measured by OD_600_, was found to be positively correlated with the expression of MK- 7 in both fermentation processes. This suggests that higher cell density leads to increased MK- 7 production. In terms of MK- 7 yield, the fermentation of immobilized cells resulted in the maximum yield of 48.33 ± 2.92 mg/L at 24 h. On the other hand, the fermentation of free cells yielded 32.76 ± 1.92 mg/L of MK- 7 but required 48 h to achieve this level of production. These findings indicate that the immobilization of cells improves the fermentation process, shortening the cycle time and increasing the yield of MK- 7 compared to free cell fermentation. Immobilization can enhance the efficiency and productivity of the fermentation process, leading to higher MK- 7 production within a shorter time frame. The membrane-bound protein MenA, recognized for its hydrophobic properties, has been extensively studied for its role in MK- 7 synthesis. Consequently, precise regulation of MenA’s structure and function can lead to the development of highly active and thermally stable enzyme variants, ultimately enhancing MK- 7 production through metabolic pathways (Hu et al. 2020; Kong and Lee 2011). There was no significant difference in the activity of the menA enzyme between free cells and immobilized cells during fermentation (Table S1). Therefore, we infer that immobilized fermentation may only affect the cell tolerance to the growth environment and the rate of material exchange, while having minimal impact on the cell own metabolic mechanisms. The potential applicability of this immobilization technology to other microbial strains may be influenced by differences in metabolic mechanisms and MenA enzyme activity among various strains.

### Comparison of continuous fermentation processes

**Fig. 3:**
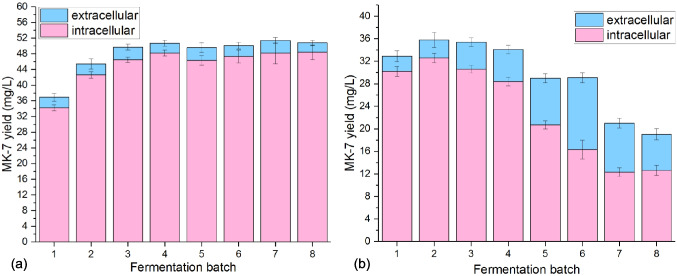
The production of MK- 7 using immobilized cells (**a**) and free cells (**b**) for eight-batch fermentations

Subsequently, the stability of fermentation was compared between immobilized cells and free cells by conducting eight consecutive batches of fermentation. It was observed that the immobilized cell fermentation maintained a stable yield of MK- 7 throughout the consecutive batches (Fig. 3a). However, in the case of free cell fermentation, there was a significant decrease in MK- 7 yield starting from the fifth batch (Fig. 3b). An interesting finding of the study was that the extracellular expression of MK- 7 in the immobilized cell fermentation accounted for only 4.7%. This suggests that a majority of MK- 7 production occurred within the cells or was retained within the immobilized structure. On the other hand, the extracellular expression of MK- 7 in the free cells increased gradually with successive batch runs, reaching 44%. These results indicated that the immobilized cells had a higher intracellular retention of MK- 7, with a lower portion being released into the fermentation medium. In contrast, the free cells exhibited a higher percentage of extracellular MK- 7, potentially due to the increased release of MK- 7 into the medium over successive batches. These findings highlight the differences in MK- 7 distribution and retention between immobilized cells and free cells during fermentation, suggesting that the immobilization technique can enhance intracellular MK- 7 retention, leading to more stable and consistent MK- 7 production across consecutive batches. After eight consecutive batches, cell density increased significantly while maintaining the structural integrity of the materials. However, the longevity of the immobilization materials needs to be assessed over extended periods in different fermentation systems and under varying mechanical agitation conditions.

The fermentation space–time yield is a crucial indicator of production capacity, which is important for reducing production costs and simplifying downstream separation and purification processes. During the validation of MK- 7 fermentation space–time yield across different experimental batches, we observed a gradual increase in the space–time yield of MK- 7 in immobilized-cell fermentation, as depicted in Figure S3. Starting from the third batch, the space–time yield stabilized at approximately 2.0 mg/L·h. In contrast, fermenting with resting cells resulted in a gradual decrease in MK- 7 concentration. From the third batch to the eighth batch, the space–time yield of MK- 7 was only 0.39 mg/L·h. This underscores the significant advantages of immobilized cell fermentation in enhancing MK- 7 yield. The above results demonstrated that continuous fermentation using remaining free cells as seeds may lead to senescence and dissolution of the cells, resulting in decreased MK- 7 production. On the other hand, the use of immobilized cells with PVA support carrier in continuous fermentation offers benefits such as enhanced production efficiency, process stability, and cost savings.

### Microscopic and SEM analysis

**Fig. 4:**
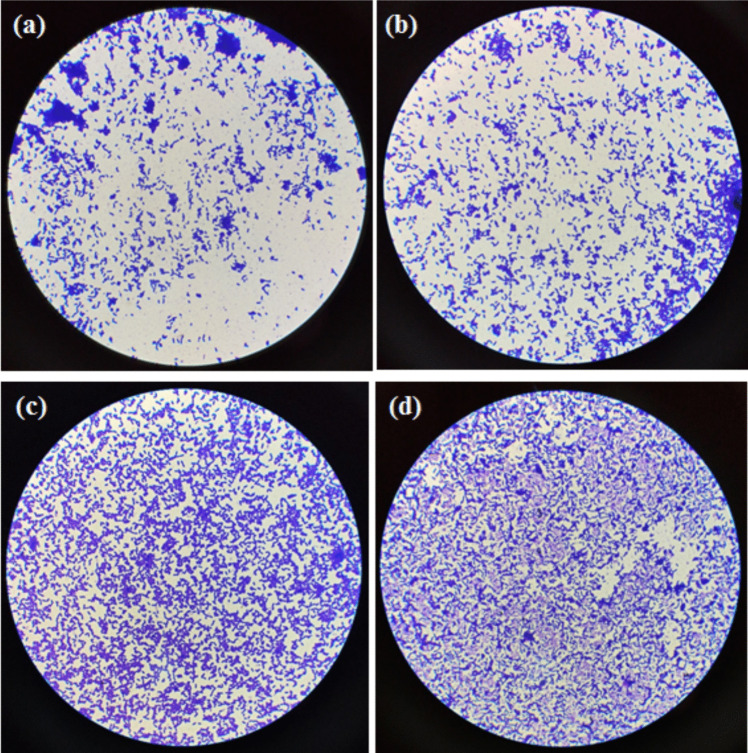
Morphology of *B. subtilis* BUCT- 184 cells. **a** Fresh cells; **b** immobilized cells after one-batch fermentations; **c** immobilized cells after eight-batch fermentations; **d** free cells after eight-batch fermentations

We further conducted microscopic investigations of the immobilized cells before and after fermentation to assess their growth status, with the results presented in Fig. 4. Similar to freshly cultured fermentation cells, we observed that immobilized cells remained predominantly active in the fermentation broth during continuous fermentation. However, after eight consecutive batches of fermentation with resting cells, the accumulation of secondary metabolites in the fermentation broth resulted in a large number of aging cells and the release of harmful substances, which adversely affected the growth of *B. subtilis* cells. Consequently, the cells transitioned into a spore form as a self-protective mechanism. The immobilized cell fermentation can provide additional protection to the cells, reducing damage from mechanical stirring and thereby better maintaining cell activity. In contrast, resting cells subjected to continuous fermentation under strong shear stirring may experience breakage, leading to the release of harmful substances, which can trigger senescence and lysis, ultimately resulting in a significant amount of extracellular MK- 7 product. This explanation has been included in the main text.

**Fig. 5:**
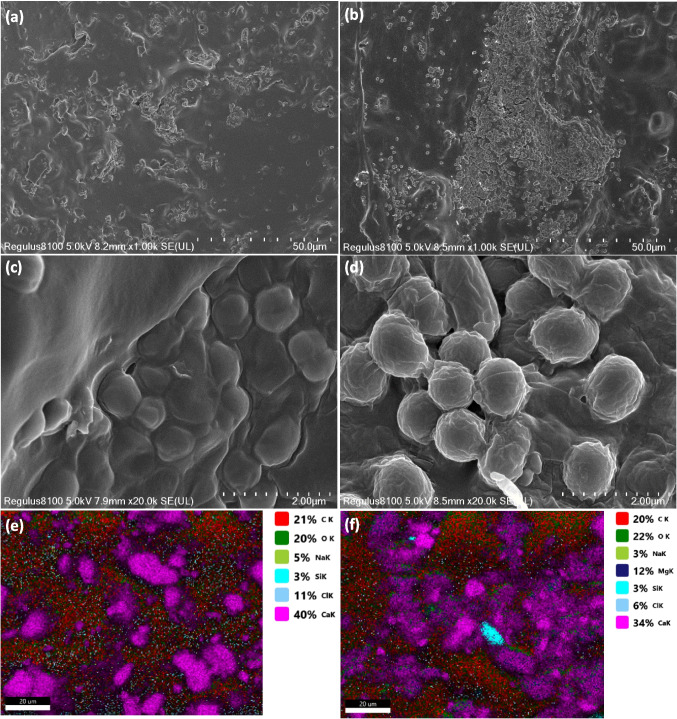
Scanning electron micrographs and spectrum-measuring of immobilized cell. **a** SEM of immobilized cell before fermentation at 50.0 μm. **b** SEM of immobilized cell after eight-batch fermentations at 50.0 μm. **c** SEM of immobilized cell before fermentation at 2.00 μm. **d** SEM of immobilized cell after eight-batch fermentations at 2.00 μm. **e** Spectrum-measuring of immobilized cell before fermentation (20.00 μm). **f** Spectrum-measuring of immobilized cell after eight-batch fermentations (20.00 μm)

Moreover, the immobilized material before and after fermentation were examined using SEM and the results are shown in Fig. 5. Compared to before fermentation (Fig. 5a), the immobilized material after fermentation shows a significant amount of biofilm attachment on its surface (Fig. 5b), indicating good compatibility between the encapsulated cells and the carrier. Various types of interactions may occur between the cell surfaces and the polymer matrix. As the cultivation time increases, the cells embedded within the carrier’s cavities continuously adsorb onto the gel surface and diffuse into the internal pores, thereby achieving a higher biomass loading. Upon further magnification, it can be observed that the cell density of the immobilized material after fermentation has significantly increased while maintaining normal cell morphology (Fig. 5d vs c). We further analyzed the surface elemental distribution of the immobilized material before and after fermentation (Fig. 5e–f). The small changes in oxygen and carbon elements suggest that the immobilized material did not undergo significant loss under mechanical stirring conditions before and after fermentation use. Meanwhile, we also observed a decrease in the distribution of Ca^2+^ in the immobilized material, from 40% before fermentation to 34% after fermentation use, which may be influenced by the covering effect of cell growth. The appearance of Mg^2+^ could be attributed to their introduction from the fermentation broth, indicating that nutrients can exchange within the material, which may be more conducive to the growth of active cells. This suggests that the immobilized material, including the cells within the PVA matrix, maintained its stability and structural integrity throughout the fermentation process.

## Discussion

By screening of appropriate hydrogel material, we developed an innovative PVA + B@Ca composite embedding strategy for immobilization of of *B. subtilis* BUCT- 184 cells, resulting in a significant improvement to a production yield of 48.33 ± 2.92 mg/L of MK- 7. This PVA + B@Ca immobilization method utilizes materials such as polyvinyl alcohol (PVA), boric acid, and calcium carbonate, all of which are relatively inexpensive. This approach can reduce fermentation time and energy consumption, as well as lower downstream separation and purification costs. Studies have shown that *B. subtilis* has a strong tendency to form films during fermentation, with biofilms forming on the surface that can potentially affect the carboxylation of the extracellular matrix and gene expression, thereby controlling the release of MK- 7 (Wu et al. 2021). The structure of PVA gel embedding, which is similar to natural soft tissue (Wang and Campbell 2009), can provide space for cell growth and favorable conditions for biofilm formation, leading to higher MK- 7 secretion. These attributes establish PVA as an ideal material for immobilized fermentation, promoting higher yields and more robust performance compared to other materials like chitosan, alginate, and gelatin.

Continuous fermentation has the potential to enhance the space–time yield, which is an important indicator of production capacity (Maxon 1960). By implementing continuous fermentation, the time required for seed cultivation, elimination, and cleaning can be significantly reduced. This leads to improved production efficiency and a more cost-effective process (Berenjian et al. 2024; Nguyen et al. 2018; Niu et al. 2025; Praveen and Loh 2015). When comparing continuous batch fermentation using immobilized porous membranes to the free cell fermentation strategy, the immobilized cells exhibited excellent stability throughout the continuous fermentation process, leading to a space–time yield of MK- 7 that reached 2.0 mg/L·h. This achievement represents a fivefold increase compared to the yield obtained using free-cell fermentation, which highlights the superiority of the immobilized porous membranes technique in enhancing fermentation performance. This unprecedented increase in productivity can be attributed to the unique protective properties of the PVA + B@Ca matrix, which creates an optimal microenvironment for cellular metabolism. The use of PVA for composite embedding in immobilized fermentation has been shown to significantly enhance stability (Gong et al. 2010), which was demonstrated through the stable productivity of MK- 7 in eight continuous batch fermentations. These findings highlighted the potential of continuous fermentation as a strategy for robust and efficient MK- 7 production. Further validation is needed to assess its feasibility for large-scale production, including adaptability to different fermentation vessels and stirring conditions, as well as cost evaluation for long-term, large-scale production. The primary limitation lies in ensuring sterile conditions during immobilization scale-up and between different fermentation batches, which is crucial for evaluating scalability benefits and costs at various scales.

Raja Mahanama conducted the first systematic study on solid-state fermentation for MK- 7 production, increasing the MK- 7 concentration from 57.78 to 67.01 mg/kg, an increase of approximately 16% (Mahanama et al. 2011b). In the face of challenges such as long fermentation times, large space requirements, and low yields, research on MK- 7 fermentation has gradually shifted toward enhancing liquid fermentation processes. Gao et al. achieved the biosynthesis of MK- 7 in *E. coli* for the first time, successfully obtaining 13.6 μM (8.8 mg/L) of MK- 7 in a 5-l fermentation tank, with a yield of 176 μg/L/h, which is a 22-fold increase compared to the initial strain (Gao et al. 2020). In the present, our findings presented in this study offer a paradigm-shifting perspective on fermentation biotechnology. The research demonstrates the extraordinary potential of immobilized fermentation produce MK- 7 using *B. subtilis* BUCT- 184, showcasing how strategic cellular embedding can revolutionize industrial MK- 7 fermentation processes.

## Supplementary Information

Below is the link to the electronic supplementary material.

Supplementary file1 (PDF 290 KB)

## Data Availability

All data supporting the findings of this study are available within the paper and its Supplementary Information.
