# Supplementary material for: Enhancement of menaquinone- 7 production through immobilization with hydrogel-based porous membranes
Source: Appl Microbiol Biotechnol. 2025 May 13;109:121. doi: 10.1007/s00253-025-13493-3 (PMC12075279; doi:10.1007/s00253-025-13493-3)
Supplement: Supplementary file 1 [file 253_2025_13493_MOESM1_ESM.pdf]

Supporting Information

Enhancement of menaquinone-7 production through immobilization with hydrogel-based porous membranes

Authors:

Qiu-Hua Zhang,[†,‡] Zheng Wang,[†] Yao-Qiang Wang,[†] Man-Lu Liu,[†] and Hai-Jia Su[†,*]

Affiliations:

[†]Beijing Key Laboratory of Bioprocess, and Beijing Advanced Innovation Center for Soft Matter Science and Engineering, Beijing University of Chemical Technology, Beijing, 100029, People's Republic of China.

[‡]Brother research center, Jiangxi Brother Pharmaceutical Co.,LTD, Jiujiang, 332700, People's Republic of China.

***Corresponding author:**

Prof. Haijia Su, E-mail: suhj@mail.buct.edu.cn

Qiu-Hua Zhang, E-mail: 360024848@qq.com

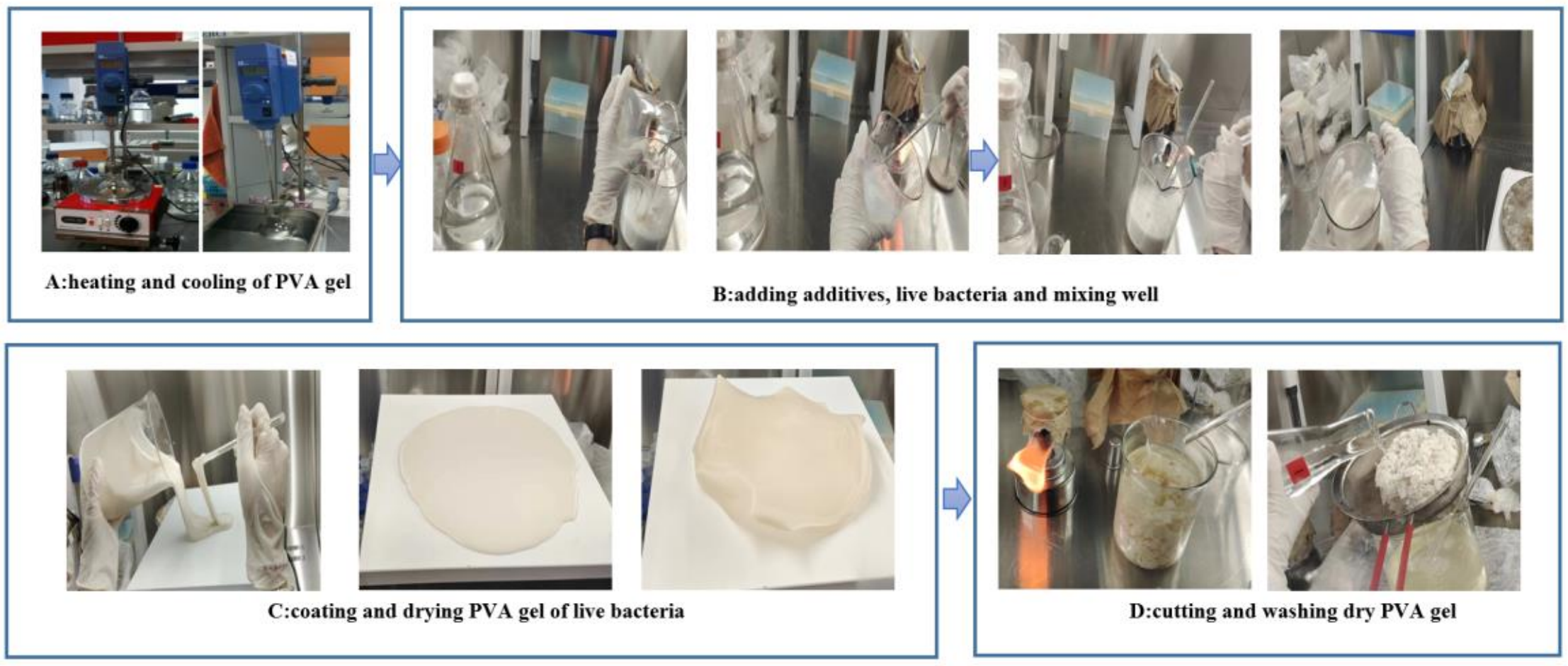

Figure. S1 The flowchart for the immobilization process.

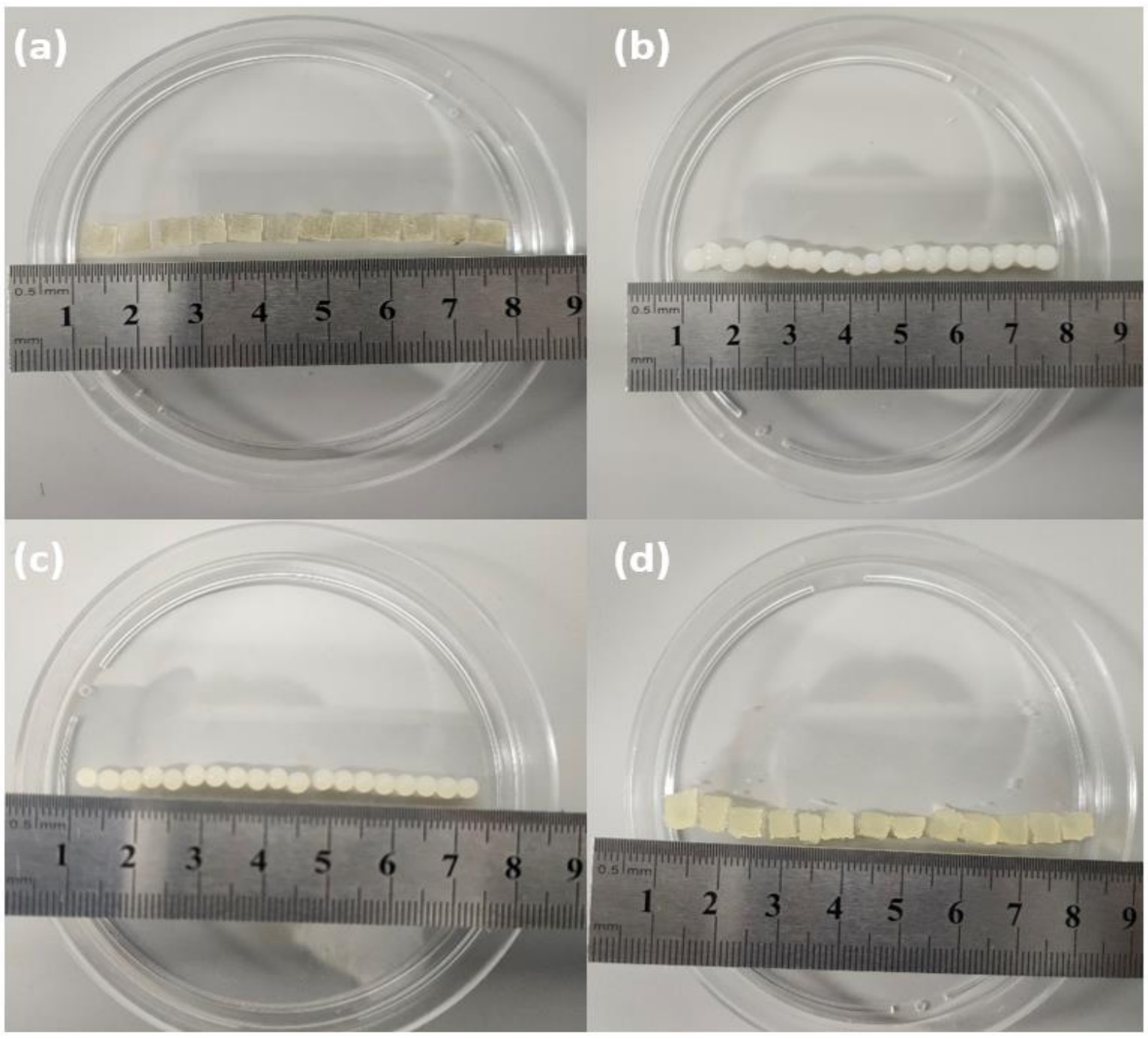

Figure. S2 Immobilized cells with different ma: Poly viny alcohol (a); Chitosan (b); Alginate (c); Gelatin (d).

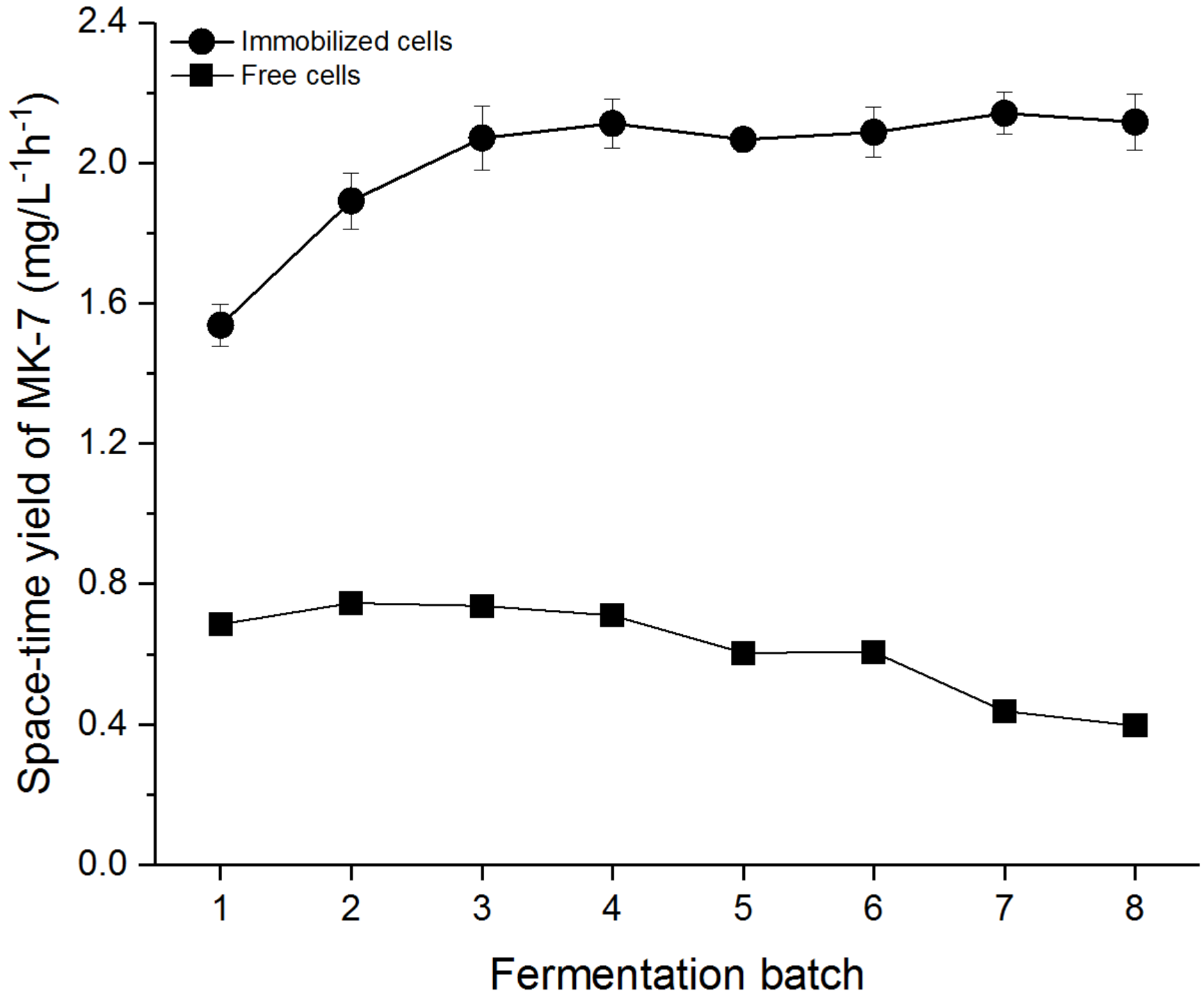

Figure. S3 Comparsion of the space-time yields of MK-7 using free cells (square symbol) and immobilized cells (circle symbol) in continuous fermentation processes.

Table S1. MK-7 production, final OD600, maximal specific growth rate (μ_{max})and maximal glycerol consumption rate ($q_{s,max}$) of Bacillus subtilis BUCT-184 strains during cultivation in 5L bioreactor.

Cells	Final OD_{600}	μ_{max} (h^{-1}) [a]	$q_{s,max}$(mg OD_{600}^{-1} h^{-1})	MK-7 yield (μg(g DCW)$^{-1}$)	Specific activity of menA[b]
Immobilized cells	16.77±0.55	1.45±0.15	32.2±3.2	161.1±8.2	0.67±0.12
Free cells	18.78±0.59	0.73±0.09	23.1±1.2	109.2±13.3	0.65±0.24

a, ΔOD_{600} per units of bacteria per hour.

b, the unit of specific activity is nanomoles per hour per milligram of protein.
